# Report of a Committee of the Boston Society, for Medical Improvement, on the Alleged Dangers Which Accompany the Inhalation of the Vapor of Sulphuric Ether

**Published:** 1861-11

**Authors:** 


### Book Notices.

Report of a Committee of the Boston Society for Medical Improvement, on the Alleged Dangers which Accompany the Inhalation of the Vapor of Sulphuric Ether. Boston: David Clapp, Printer. 1861.

Boston's bantling, Sulphuric Ether, is in danger of being nursed to death. Our Committee has endeavored to prove too much. And, as in all cases of over-zealous care, the object of the solicitude must suffer.

When Drs. Hodges, Hayward, Townsend, Jackson and Upham, the gentlemen of the above Committee, were appointed to report on "the alleged dangers" etc., we believed that this moot question would be definitely and satisfactorily settled, and we looked for the publication of their report with no little interest. For, notwithstanding the notorious Bostonian predilection for its own form of the boon, the discovery of anæsthesia gave to suffering humanity, the eminent names composing the Committee,—names which have for years graced the annals of American medical science, in its every branch,—were to us, a guaranty that all that intelligent research, philosophical argument and indefatigable industry could accomplish, would be given to the solution of the problem: *Is the production of insensibility, by the vapor of Sulphuric Ether dangerous?*

4

If this question is answered at all, in the Report under consideration, we leave it to our readers to judge, from an impartial review of its main features, how far that answer is in the negative;—hesitating, for ourselves, only as we would not pre-judge the case, to say that to our mind the capabilities of the vapor of sulphuric ether for dangerous results and complications, have never before been so strongly presented. But our readers shall judge for themselves.

Passing over the not inconsiderable portion of the Report, which discusses the comparative merits of ether and chloroform,—it is to the analysis of alleged deaths from ether we take exceptions. The Committee have gathered, by extensive correspondence, newspaper and journal notices, and careful scrutiny of the periodical literature since Dr. Morton's discovery, every instance (41) of alleged fatal result from the inhalation of ether. We subjoin for comment, the following cases:

Case 1. Hotel Dieu of Auxerre, France, 1847. A man, fifty-five years old, after having breathed sulphuric ether, was operated on for cancer of the breast, and died during the operation, with evident symptoms of asphyxia. The ether was inhaled from a Charrière's apparatus. "The want of care in administering the ether, which was given in a manner likely to produce asphyxia, and the insufficient means used for the restoration of the patient, sufficiently explain the cause of death."—*Exposition et Histoire des Principâles découvertes Scientifiques modernes*, par Louis Figuier, Paris, 1851, t. ii., p. 282.—*Gaz. Médicale*, 4 Mars, 1845, p. 170.

Case 16. Dr. C. P. Johnson, Richmond, Va., 1853. A negress of middle age, had, three months previously, undergone resection of the parts adjoining the symphysis of the lower jaw, for an osteoid cancer of the parts. The disease re-appeared and rapidly invaded the neighboring structures, involving a considerable portion of the remnants of the body and ramus of each side. The patient's appearance was cachectic. A complete disarticulation was decided upon, and sulphuric ether was administered. The operation was tedious. After the separation of the diseased mass, the branches of the internal maxillaries gave so much trouble, that one, if not both, carotids were tied. The regularity of the patient's stertorous breathing was not interrupted till just before the close of the operation. As soon as this was noticed, some brandy and water was passed into the fauces, but was regurgitated with the production of one or two spasmodic actions about the glottis, which were the last vital manifestations that occurred. Artificial respiration, friction, etc., induced no response.

The tissues were found ex-sanguine at the autopsy.—*Committee's Correspondence.*

Case 19. M. Barriere, Lyons, France, 1852. A female, aged fifty-five, in a weak and bad state of general health, underwent excision of the superior maxillary bone. Sulphuric ether was given from a sponge placed in a bladder. Death occurred during the operation. It was thought possible that it might have been due to the hemorrhage.—*Gazette des Hopitaux*, June 18, 1853.

Case 20. Dr. G. de Oettingen, Dorpat, Russia, 1847. Constantly increasing anæmia, on account of a gangrenous ulcer of the leg, rendered amputation necessary in the case of an old man, seventy years of age. Sulphuric ether was administered from a common bottle, having an opening large enough to include the nose and mouth of the patient. The operation was completed, but the arteries were not tied when the indications of approaching death were noticed, and which shortly occurred with symptoms of syncope. There was no hemorrhage.

The autopsy gave no explanation of the death.—*Committee's Correspondence.*

Case 23. Dr. ———, New York, 1860. "A patient with hernia had been laboring under symptoms of strangulation for some time, and was in a desperate condition. A cutting operation for his relief was resolved upon. He was fully anæsthetized with sulphuric ether, and suddenly, during the progress of the operation, showed symptoms of prostration, and soon died."—*Committee's Correspondence.*

Case 25. Dr. ———, New York, 1860. "A very large, old, scrotal hernia, had, from some cause, become irreducible. Inhalation of sulphuric ether was resorted to, and, while the patient was under its full influence, the hips being raised, and the head allowed to be forcibly flexed upon the chest, the taxis was resorted to. The large mass of intestines very suddenly receded into the abdomen, and just at the moment the patient was noticed to be in a dying condition, from which he could not be recovered."—*Committee's Correspondence.*

It is intimated that too little attention was probably given to the state of the respiration and the pulse. The absence of an autopsy in this instance is greatly to be regretted.

Case 27. Dr. J. Y. Bassett, Alabama, 1847. It was proposed to apply the actual cautery in a case of tetanus. Sulphuric ether was administered by a dentist. "At this time the patient's pulse was good, and there was no signs of an immediate extinction of life. In one minute the patient was under its influence; in a quarter more he was dead—beyond all my efforts to produce artificial respiration or to restore life. All present thought he died from inhaling ether."—*Amer. Jour. Med. Science*, Vol. 18, p. 293.

In cases 1 and 25, the deaths are alleged to be due to *asphyxia*, and the Committee claim that it (the asphyxia) was "brought about by wholly avoidable causes,"—which allegation and claim is also made in two other cases we do not quote. In Nos. 16, 19 and 20, "the character and circumstances of the operation certainly absolve the ether" (p. 11, of *Report*). In Nos. 23 and 27, the disease is alleged to be the cause of death. In twenty-three cases, death occurred within twenty-four hours, in seventeen of them, "death was immediate or nearly so" (*idem*). In the whole forty-one cases, the "probable causes" of death assigned are as follows: In four, asphyxia; in twelve, shock of accident or operation; exhaustion; shock, complicated with exhaustion, with age, with general health, with hemorrhage, etc.; in one each, pneumonia, pleuritis, bronchitis, erysipelas and bronchitis, capillary bronchitis, intense local inflammation, comminuted fracture ossa innominata and perhaps, fatty degeneration of heart, disease of the brain, delirium tremens, arachnitis, symptoms of meningitis, acute pulmonary disease; in two each, pyæmia, tetanus; and in nine cases no "probable cause of death" is assigned in the table, but in the body of the report (p. 10) we find three of them are excluded from consideration under the rule, that the death "must occur while the patient is actually in an anæsthetic state,"—the time of death varying from three to fifteen days in these three cases; two others (of these nine) viz: Nos. 19 and 23, are cited above; of another, "the details are very meagre, but that the death [occurring four hours after inhalation] had any connection with the anæsthetic is, at least, very improbable;" and we suppose the same plea may be set up for the remaining three cases.

On page 12, of their report, the Committee aver, that "their careful search of journals and monographs, furnishes not a single conclusive case of death from the proper inhalation of pure sulphuric ether." In view of the above summary of cases reported, and yet with all deference to the Committee, we submit that with such latitude as is taken in their analysis, the most careful search of journals and monographs would furnish not a single conclusive case of death from the proper inhalation of pure *chloroform.* Of the attack on this latter agent, we have nothing to say, except, that the mode of argument, of which the following is a specimen, hardly convinces us, more fully, that the epithets "*fleau chloroformique*," "suspended sword," etc., are deserved, than the report itself does that ether, no matter how cautiously administered, is entirely innocuous :—

"But whether this is true or not, that 30,000 soldiers should escape the dangers of chloroform, is no argument in its favor. It is well known that a vast number of missiles are thrown in battle without harming a single person, yet no one would pretend that this fact diminishes in the slightest degree, the danger in the flight of a solitary bullet. The position of chloroform is precisely identical."

"Identical" with this difference, that if the vast number of missiles thrown in battle were directly applied to the soldiers, in such a way as to produce their legitimate effects, (in other words, *hit them*), as the chloroform was to the 30,000, and the soldiers yet received no injury from them, the degree of danger in the flight of each bullet would scarce warrant Gen. McClellan in relying upon them to subdue the rebellion.

One more word, and we have done fault-finding: Of the forty-one deaths reported, eighteen are stated to have occurred at intervals, varying from forty hours to sixteen days after the inhalation ; seven, in from fifteen or twenty minutes to fifteen hours ; four, during the operation ; four, during the inhalation ; and in the remaining cases, the intervals are not given. On page 10, we find the two following conditions laid down as essential to the maintainance of a charge of fatal result, attributable to the anæsthetic :

"1st. That the event should occur while the patient is actually in an anæsthetic state."

2d. That the circumstances of its occurrence should be inexplicable by any phenomena of the disease or operation."

Under the first rule, only eight cases out of the forty-one merit attention. Yet the Committee have abrogated the rule in twenty-one additional cases, where some other of the list of causes of death, aside from the anæsthetic, could be given; have availed themselves of it in four cases, where none of those causes is assigned; and in eight cases, where the date of death is not given, but is assumed to be "immediate or nearly so," they have assigned causes in five cases, and of the remaining three, remark that "the connection between the result and the inhalation is either problematical, or else manifestly absurd and unfounded," (p. 11.) On the whole, a mode of investigation not eminently satisfactory to the mathematical mind.

The incidental practical hints in the report are invaluable, and we quote as much of them as we have space for. Absolute purity of the ether for inhalation is insisted upon; and the brands of Powers & Weightman, of Philadelphia, and Dr. Squibb, of Brooklyn, N. Y., are commended as uniformly of excellent quality. A new stiff towel, of pretty good size, folded to about ten inches by five, and the ends lapped over, and secured by pins, so as to form a cone, is suggested as the best medium for its administration.

* * * The thick layers of towelling will hold sufficient ether, and its texture prevent a too free dilution of the anæsthetic by the atmospheric air, provided the apex and seam of the cone are carefully and tightly closed, either by pins or the fingers. As the cone becomes collapsed by saturation, it should from time to time be opened, and kept in shape by distending it with the hand. Unless these details are attended to, and especially the closure of the apex of the cone, the induction of anæsthesia will be uncertain and protracted. In anything so porous as a towel or sponge, the difficulty is to exclude enough air; for while its adequate admission to the lungs during etherization is essential to the life of the patient, its too free entrance not only delays anæsthesia, but induces a condition of excitement, both mental and physical. The importance of excluding the air, as above stated, is a point not generally appreciated, but the necessity of it has long been known to those most accustomed to the use of ether, as shown by the "chemise" with which, in hospital practice, a too porous sponge is often covered to expedite the etherization of a rebellious patient.

Ether should be poured lavishly on the towel or sponge, an ounce or two at a time, especially at the commencement of inhalation. Although it may be wasted, too much, so far as safety is concerned, cannot be used. A small quantity poured on hesitatingly and timidly, as is sometimes done, has the same effect as a too free dilution of the vapor with air, producing simply intoxication and its accompanying excitement, without anæsthesia; whereas, a large amount, though the cough and choking sensation, which the greater volume of vapor produces, may cause the patient to resist and struggle, is certain to bring about a satisfactory condition of insensibility.

* * * When there is muscular rigidity with lividity, the suspension of etherization will transform this into the relaxation of anæsthesia. * * * The pulse should be watched by a competent person from the outset, and its failure, either in strength or frequency, lead to a more cautious use of the ether. * * * The respiratory movements should not be forgotten or neglected, but any slowness or irregularity in their performance should at once receive attention. * * * In the pulse of the healthy subject suddenly reduced by accident, the vitality is unimpaired, and the pulse even when hardly perceptible, rises with anæsthesia. Ether, therefore, is not to be withheld from the patient to be operated on, even in a state of collapse after a severe accident, but great caution is demanded in its use with patients who are near death from chronic and exhausting disease, and who require operations.*

* * * The best test of complete etherization is the snoring of the patient; and no operation, unless slight, should be undertaken, until this symptom presents itself. * * * There is, * * * a condition prone to manifest itself with children, especially those who are weak, strumous or overgrown, which is due to the cumulative properties of ether. It may show itself after almost any degree of etherization, and is characterized by a feeble pulse and slow respiration, not passing off with the readiness usually marking the phenomena of etherization. With young persons, a cautious inhalation of five minutes will often induce an anæsthesia of half an hour, an effect wholly out of proportion to that which the same amount

*Unpublished Records of the Boston Society for Med. Improvement.

of ether would produce in an adult. This state is not a dangerous one, and only requires time to dissipate its symptoms. Compression of the chest will expel the fumes of ether being eliminated from the pulmonary surface, and admit the entrance of a fresh supply of oxygen to stimulate the circulation. The inhalation should therefore be suspended at short intervals with children, and but little ether given at a time. Undoubtedly, it should also be used cautiously with persons past the middle period of life, of such a general obesity or constitutional condition as may lead to the supposition of a fatty degeneration of the heart. In none of the alleged deaths from ether, however, is there any mention of valvular disease of the heart being found. Of this, then, and of any bad effect upon pulmonary affections, there need be no fear, for we see it constantly administered without detriment to persons more or less advanced in phthisis, for the common operation of fistula in ano.

All that is as yet positively known of the physiology of anæsthesia is given as follows, from the recent, elaborate treatise of Lallemand, Perrin and Duroy:

1st. Anæsthetics are neither transformed or destroyed in the system, but are rapidly eliminated from it, chiefly by the lungs, and to a limited extent by the cutaneous surface. Chloroform and amylene being insoluble in water, no traces of them are ever found in the urine. Sulphuric ether being more soluble, a small quantity of this may be detected by reagents in the renal secretion.

2d. The blood and the organs of animals, dead from *etherism* (the name given to this special intoxication by the above-named authors), contain the anæsthetic agent employed, the presence of which is easily determined by special chemical research; and the following figures show in what proportion the principal viscera contain the anæsthetic, in reference, for each of the agents employed, to the quantity found in the blood:

| Source of Analysis. | Chloroform. | Sulph. Ether. | Amylene. |
|---|---|---|---|
| Blood,................ | 1.00 | 1 00 | 1.00 |
| Cetebral substance,.... | 3.92 | 3.25 | 2.06 |
| Liver,................ | 2.08 | 2.25 | 1.00 |
| Muscular tissues,....... | 0.16 | 0.25 | Traces. |

From this, it appears that anæsthetics accumulate in the cerebro-spinal system.

3d. It is not easy to explain the deaths from chloroform and amylene, or from ether (as sometimes seen in the lower animals), but it would seem probable, both from the phenomena which they present, and the experiments which have been made, that they are the consequence of an abolition of the functions of the nervous system, and not of asphyxia.

The general conclusions which have been arrived at by the Committee, are summed up as follows:

1st. The ultimate effects of all anæsthetics show that they are depressing agents. This is indicated both by their symptoms and by the results of experiments. No anæsthetic should therefore be used carelessly, nor can it be administered without risk, by an incompetent person.

2d. It is now widely conceded, both in this country and in Europe, that sulphuric ether is safer than any other anæsthetic, and this conviction is gradually gaining ground.

3d. Proper precaution being taken, sulphuric ether will produce entire insensibility in all cases, and no anæsthetic requires so few precautions in its use.

4th. There is no recorded case of death, known to the Committee, attributed to sulphuric ether, which cannot be explained on some other ground equally plausible, or in which, if it were possible to repeat the experiment, insensibility could not have been produced, and death avoided. This cannot be said of chloroform.

5th. In view of all these facts, the use of ether in armies, to the extent which its bulk will permit, ought to be obligatory, at least, in a moral point of view.

6th. The advantages of chloroform are exclusively those of convenience. Its dangers are not averted by its admixture with sulphuric ether in any proportions. The combination of these two agents cannot be too strongly denounced as a treacherous and dangerous compound. Chloric ether, being a solution of chloroform in alcohol, merits the same condemnation.

Dr. C. T. Jackson, one of the Committee, objects and excepts to the clause in this report in which "all mixtures of ether and chloroform" are denounced; viz: to the words, "the dangers of chloroform are not averted by admixture with sulphuric ether," and to the terms, "treacherous and dangerous compound" of ether and chloroform. He believes that a mixture of four measures of ether and one measure of chloroform may be employed without danger, or with very little danger, and that the risks from chloroform are diminished more than four-fifths by this combination. He believes it to be necessary to have an anæsthetic agent of less bulk than ether, and not so dangerous as chloroform, for army uses, and is satisfied that this mixture, which he has employed and prescribed, completely answers the purpose required.

